# Structural Properties of Coniferyl Alcohol-Based Low Transition Temperature Mixtures

**DOI:** 10.1021/acsomega.5c08613

**Published:** 2026-01-29

**Authors:** Kosuke Ikeda, Takumi Karasawa, Takeki Miyazawa, Yoshiki Horikawa, Kento Kimura, Yoichi Tominaga, Toshiyo Kato, Yasuyuki Matsushita

**Affiliations:** † Institute of Agriculture, 13125 Tokyo University of Agriculture and Technology, 3-5-8 Saiwai-cho, Fuchu, Tokyo 183-8509, Japan; ‡ Institute of Engineering, Tokyo University of Agriculture and Technology, 2-24-16 Naka-cho, Koganei, Tokyo 184-8588, Japan; § Faculty of Engineering, Tokyo University of Agriculture and Technology, 2-24-16 Naka-cho, Koganei, Tokyo 184-8588, Japan; ∥ Smart-Core-Facility Promotion Organization, Tokyo University of Agriculture and Technology, 2-24-16 Naka-cho, Koganei, Tokyo 184-8588, Japan

## Abstract

This study examines the structural organization of low transition temperature mixtures (LTTMs) based on coniferyl alcohol, a primary monolignol, at the macroscopic and microscopic levels. The work presents the first investigation into the use of lignin-derived monomers as constituents of LTTMs. The LTTMs prepared with 2:1 and 3:1 molar ratios of coniferyl alcohol to choline chloride exhibited glass transition temperatures (*T*_g_) of −24.6 and −16.8 °C, respectively, and with no observable melting points (*T*_m_). The respective water contents were 0.4790 and 0.7564 wt %, similar to that reported for other natural deep eutectic solvents. Time–domain nuclear magnetic resonance analysis revealed that these LTTMs are heterogeneous and exhibited two regions between 20 and 90 °C due to differences in molecular mobility. Furthermore, Fourier-transform infrared spectroscopy and proton NMR demonstrated that the region of restricted molecular mobility was stabilized by strong hydrogen bonding, mainly involving the γ-OH group of the coniferyl alcohol. The weak hydrogen bonds were gradually broken with an increase in temperature, leading to structural reorganization. As the temperature increased, the system transitioned to a state in which the hydrogen-bonded clusters and more freely dispersed molecular regions were present in approximately equal proportions.

## Graphical abstract

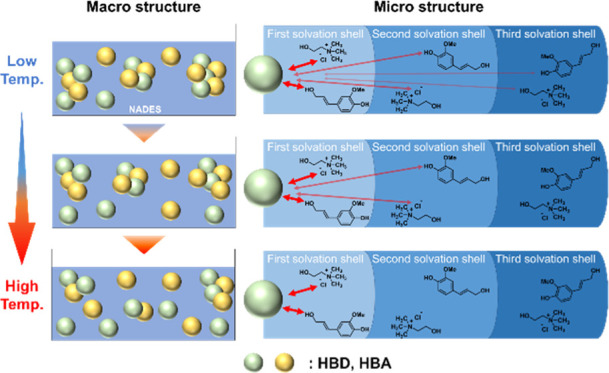

## Introduction

Deep eutectic solvents (DES) are generally defined—though the definition remains debated,^1^—as mixtures of hydrogen bond acceptors (HBAs) and hydrogen bond donors (HBDs) in selected proportions whose mixture has a melting point (*T*_m_) lower than those of the pure components. The archetypal example is the 2:1 urea/choline chloride (ChCl) system reported by Abbott et al.^2^ (*T*_m_urea_ = 133 °C; *T*_m_ChCl_ = 302 °C; eutectic at 12 °C). In 2012, Francisco et al. introduced low transition temperature mixtures (LTTMs),^3^ discussed here as an operational subclass of DES, that exhibit a glass transition temperature (*T*_g_) rather than *T*_m_. In this work, we adopt this terminology in what follows, while acknowledging that DES and LTTMs share similar characteristics and are sometimes discussed interchangeably.^3^

DES and LTTMs are closely related and share features such as low vapor pressure, low toxicity, nonflammability, biocompatibility, and biodegradability.^3,4^ However, LTTMs generally remain liquid over a wider temperature window:^5^ their *T*_g_ are often below 0 °C,^3^ and are typically lower than the *T*_m_ observed for many DES formulations.^4^ Consequently, LTTMs can function as solvents across broader temperature ranges than DES. Reflecting these properties, LTTMs have been used for lignocellulosic biomass processing,^5^ natural-product extraction,^6–9^ as solvents for chemical synthesis,^10–13^ and as CO_2_ absorbents.^14–16^

Understanding the structure of DES and LTTMs is essential for comprehending reaction mechanisms, such as extraction and adsorption, improving reaction efficiency, and enhancing their value as green solvents like ionic liquids. Formation of these liquids is controlled by the strength of the hydrogen-bond network.^17^ Key variables are (i) relative acidity/basicity of HBA/HBD and their functional groups (−OH, –COOH, and –CONH_2_),^4^ (ii) the mixing ratio,^18^ (iii) the amount of water and trace impurities,^19,20^ and (iv) molecular structure (size, symmetry, charge distribution, and aromatic/π interactions).^21,22^ Prior studies on these solvents have therefore focused on hydrogen bonding and the interactions between the HBAs and HBDs, employing theoretical simulations,^23^ and techniques such as neutron scattering^24^ and Fourier-transform infrared (FT-IR)^25^ to reveal their microstructures, and nuclear magnetic resonance (NMR) spectroscopy to elucidate their supramolecular structures. Abbott et al. used heteronuclear Overhauser effect spectroscopy (HOESY) to find correlations between the fluoride ions and urea protons in a ChCl-urea (1:2) DES.^2^ Choi et al. demonstrated, using HOESY (NOESY), that some protons in a sucrose-malic acid DES interact and aggregate into a larger structure.^26^ Delso et al. reported a ternary system consisting of ChCl-urea/glycerol/ethylene glycol-water, supporting the formation of supramolecular structures.^27^ Similarly, the LTTM structures have been investigated. Using NOESY, Hussin et al. reported hydrogen-bond interactions between the hydroxyl groups of DL-menthol and those of three different HBAs (Thymol, Sesamol, and 3-hydroxyl benzoic acid); water made a significant contribution, and water-mediated hydrogen bonds interconnecting hydroxyl groups were inferred.^28^ Carpin et al. used NOESY to observe water-content-dependent changes in the supramolecular organization of a fructose–glycerol–water ternary LTTMs.^29^ However, the uniformity of the supramolecular structure has not yet been sufficiently investigated.

This study aims to analyze the uniformity of the supramolecular structures in LTTMs. Herein, LTTMs were prepared using coniferyl alcohol (CA), a primary monolignol comprising lignin, as an HBD, representing an initial step in utilizing lignin as one of the most abundant biomass resources. ChCl, a low-cost, low-toxicity, biodegradable, biocompatible compound known as vitamin B2 complex,^30^ was used as an HBA. The macro- and microstructural properties of the prepared LTTMs were investigated using various techniques.

## Experimental Section

### Materials

ChCl (>95%) and ethanol (>99.5%) were purchased from FUJIFILM Wako Pure Chemical and Kanto Chemical, respectively. ChCl was vacuum-dried at 50 °C for at least 1 day to eliminate moisture because it is hygroscopic. CA (>92%) was synthesized according to a previously reported method.^31^ The structure and purity of CA were confirmed using ^1^H NMR (JEOL, JNM-ECA600, 600 MHz, Figure S1) and gas chromatography–mass spectrometry (GCMS, Shimadzu, GCMS-QP2020 NX, Figure S2), respectively.

### Preparation of the Samples

The samples were prepared by the method proposed by Florindo et al.^30^ The ChCl (HBAs) and CA (HBD) were weighed in molar ratios of 1:3, 1:2, 1:1, 2:1, and 3:1, ground in a mortar with a pestle at 25 °C, followed by drying in a vacuum oven (Yamato, ADP200) at 50 °C for at least 1 day to remove moisture. The dried mixtures were labeled as listed in Table 1 and used for all analyses.

**Table 1:** Sample Codes Prepared for This Study

HBAs	molar ratio (CA/HBAs)	sample codes
	1:3	E1
	1:2	E2
ChCl	1:1	E3
	2:1	E4
	3:1	E5

### Mixture Type Determination: DESs vs LTTMs vs Other

The thermal properties of the mixtures were investigated by using differential scanning calorimetry (DSC, Hitachi High-Tech Science, DSC7020) to classify their solvent types. The samples were heated from 25 to 100 °C during the first step, cooled to −100 °C, and then heated again to 100 °C during the final step. Both heating and cooling were performed at a constant rate of 10 °C min^–1^. The measurements were conducted under a nitrogen gas flow of 40 mL min^–1^. The *T*_m_ and *T*_g_ values were determined from the second heating curve. Also, the water content of the LTTMs was measured by the Karl Fischer titration method (Mitsubishi Chemical Analytech, Moisture Meter CA-200) after dissolution in ethanol. Each value represents the average of three experiments, and the error was expressed as the standard deviation.

### Macro Structural Analysis of LTTMs

The E4 and E5 macrostructures were analyzed by measuring the average proton mobility through time domain NMR (TD-NMR, Bruker Biospin, minispec mq20, 20 MHz). Each sample was filled in a tube to a height of approximately 1 cm and was heated from 20 to 90 °C in 10 °C increments. The solid echo method was applied at 20–40 °C and the Carr–Purcell–Meiboom–Gill (CPMG) method at 50–90 °C. The number of components in the supramolecular structure of the LTTMs was determined by comparing the experimentally obtained relaxation curve data, especially the transverse relaxation time (*T*_2_) curves, with the theoretical fitting data. In TD-NMR, *T*_2_ is governed mainly by ^1^H–^1^H dipolar interactions. When molecular mobility increases, these interactions are effectively lower and *T*_2_ becomes longer; when mobility decreases, the interactions are stronger and *T*_2_ becomes shorter. The fitting was performed using the following equation: $f\left(t\right)=\sum_{i=1}^{N}A\left(i\right)\exp{\left\{-\frac{1}{W\left(i\right)}\left(\frac{t}{T_{2}\left(i\right)}\right)\right\}}^{W\left(i\right)}$ , where *N* is the number of components, t is observation time, and *A*(*i*), *W*(*i*), and *T*_2_ (*i*) are the ratio, Weibull parameter, and *T*_2_ of the ith component, respectively. The temperature variation in the *T*_2_ and ratio of each component derived from the fitting was calculated. The *T*_2_ curves were measured three times for each sample.

### Microstructural Analysis of LTTMs

The LTTMs microstructures were analyzed using FT-IR (PerkinElmer, Spectrum Frontier) with the attenuated total reflection (ATR, diamond prism) mode and ^1^H NMR (Figures S3a and S6a). The FT-IR spectra were recorded at 4000–400 cm^–1^ with a resolution of 4 cm^–1^ at 25 °C, averaging eight scans per sample and corrected using the ATR correction function of the software provided by the manufacturer. For the ^1^H NMR measurements, the samples were dissolved in dimethyl sulfoxide-*d*_6_ (DMSO-*d*_6_, Cambridge Isotope Laboratories, including 0.05% v/v tetramethylsilane, TMS) to achieve a E4 and E5 concentration of 50 wt % and filled in the outer tube of a 5 mm Φ coaxial NMR tube. The inner tube was filled with DMSO-*d*_6_. The spectra were recorded at 25 °C and at 10 °C increments from 30 to 90 °C. The number of scans was set to eight, and the bands were assigned using ^13^C (Figures S3b, S6b), heteronuclear single quantum coherence (HSQC, Figures S4 and S7), and correlation spectroscopy (COSY) spectra (Figures S5 and S8). Each measurement, both FT-IR and NMR, was performed three times, and the errors were expressed as standard deviations.

## Results and Discussion

### Solvent-Type Classification

The *T*_m_ and *T*_g_ values of E1–5 are shown in Table 2 and Figure S9. The *T*m values were obtained only for the E1–3. The enthalpies of fusion (Δ*H*) of E1–3 were 56 ± 2, 38 ± 4, and 3 ± 1 mJ mg^–1^, respectively. However, E1–E3 cannot be classified as DES, since their *T*m values are not lower than those of both neat components (HBA and HBD). In contrast, E4 and E5 can be regarded as LTTMs based on their defining thermal behavior. Based on the *T*_m_ observation, subsequent experiments focused exclusively on E4 and E5, which, lacking a distinct *T*_m_, can serve as effective solvents across a wider thermal window than DESs due to their ability to remain liquid at more varied temperatures.^5^

**Table 2:** *T*_m_ and *T*_g_ Values of E1–5 and CA

sample code	*T*_m_ (°C)	*T*_g_ (°C)
E1	77 ± 0	–30 ± 1
E2	76 ± 0	–34 ± 0
E3	74 ± 1	–41 ± 11
E4		–25 ± 0
E5		–17 ± 8
CA	70.1 ± 0	

### Water Content in LTTMs

The measured water contents of E4 and E5 were 0.4790 ± 0.0006 and 0.7564 ± 0.0003 wt %, respectively, similar to those reported for other DES in previous studies.^27,32^ In general, the DES hygroscopicity is influenced by the presence of hydrophilic groups,^33^ and the effect of such groups has been studied extensively.^30,34,35^ Therefore, the higher hygroscopicity in E5 is attributed to its higher –OH group concentration (1.5 times that of E4).

### Macrostructural Analysis of LTTMs Using TD-NMR

For the TD-NMR analysis, the protons originating from water were negligible because of the very low water content in the samples (Table S1). Here, shorter *T*_2_ indicates lower mobility/stronger ^1^H–^1^H interactions, whereas longer *T*_2_ suggests higher mobility/weaker effective interactions. Accordingly, the TD-NMR response contains contributions from more-mobile and less-mobile proton populations, reflecting heterogeneity in interaction strength rather than assignment of each molecule. The fitting of the obtained data, assuming that LTTMs consist of one to three components, reveals the presence of a two-component system in these LTTMs at all temperatures, with different *T*_2_ values (Figure 1a,b). In Figure 1, the first component (first) represents a constituent with a shorter *T*_2_ and lower molecular mobility, while the second component (second) shows a longer *T*_2_ and higher mobility. The existence of two distinct *T*_2_ indicates the presence of two regions: one with strong interactions and low mobility and the other with weak interactions and high mobility. Previous studies have shown that DES form supramolecular structures,^27,36^ and are stable up to a 50 wt % water content.^19,37,38^ In accordance with this observation, it is considered that E4 and E5 similarly retain their supramolecular structures. The presence of the two distinct *T*_2_ components may suggest the presence of heterogeneous structures in the two-component system. This heterogeneity can be attributed to the variation in the strength of the hydrogen bonds.^39–43^ In both samples, the ratios of the two components exhibited similar behavior, with the low-mobility region occupying a high ratio and the high-mobility region occupying a low ratio at room temperature. However, as the temperature increases, these ratios converge to approximately the same value. This behavior may indicate temperature-dependent changes in their supramolecular structures.

**Figure 1:**
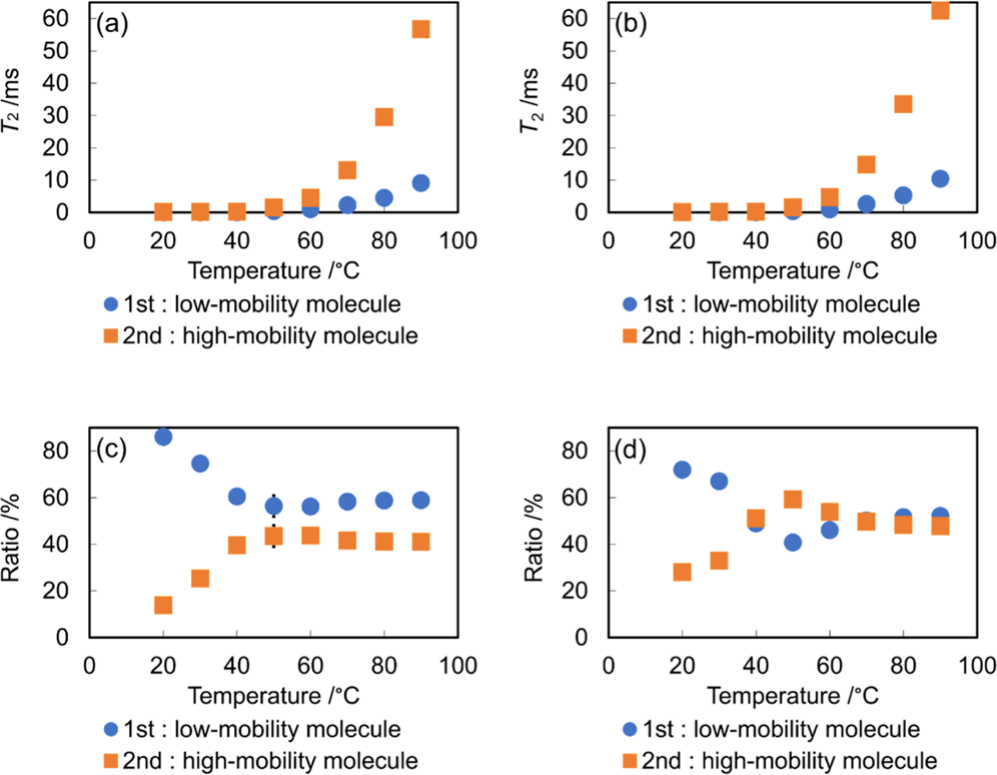
Temperature-dependence of *T*_2_ of (a) E5 and (b) E4. The temperature dependence of the ratio of the two components in (c) E5 and (d) E4.

### Microstructural Analysis of LTTMs Using FT-IR and ^1^H NMR

As shown in Figure 2, the blue, red, green, and yellow lines correspond to E5, E4, neat CA, and ChCl, respectively. The FT-IR spectra of the samples reveal the disappearance of the band corresponding to the –OH group (3460 cm^–1^) of CA (Figure 2a), suggesting the generation of a new hydrogen bond during the LTTMs formation.^44^ Furthermore, the spectrum of E5 exhibits a larger red-shift of the –OH group than that of E4, suggesting that E5 formed stronger hydrogen bonds (Figure S10). No significant changes were observed for the bands corresponding to the aromatic ring C–C bonds (at 1594 and 1515 cm^–1^, Figure 2b), indicating that the π-electrons were not involved in the LTTM formation. The bands assigned to the aromatic C–H bond in the 900–650 cm^–1^ region (Figure 2c) implied that these bonds are likely to contribute to LTTMs formation. (CA: 854 cm^–1^, E4:861 cm^–1^, E5:860 cm^–1^, and CA: 769 cm^–1^, E4:755 cm^–1^, and E5:755 cm^–1^).

**Figure 2:**
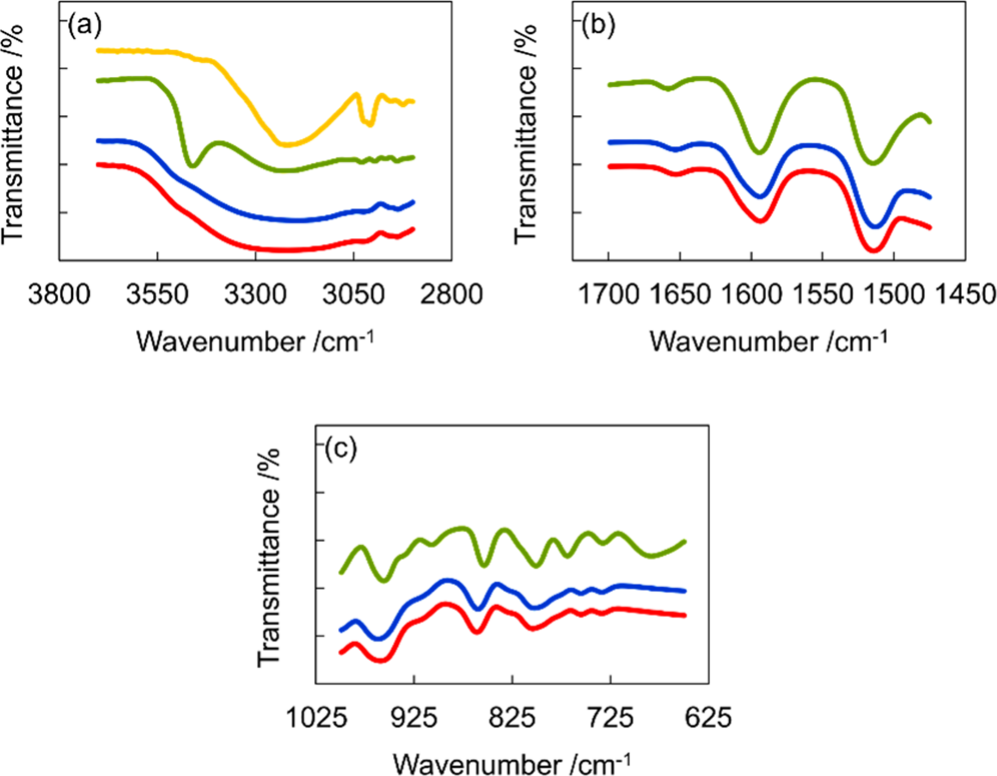
FT-IR spectra of E5 (blue), E4 (red), neat CA (green), and neat ChCl (yellow) in the regions corresponding to (a) –OH groups, (b) C–C bond in the aromatic ring, and (c) C–H bond in the aromatic ring.

For a more comprehensive analysis to elucidate the hydrogen bonding establishment, ^1^H NMR measurements were performed. The Δδ values depicted in Figure 3 are the differences between the chemical shifts of the LTTM samples at a given temperature and those of neat CA or ChCl in DMSO-*d*_6_ at 25 °C. The observed Δδ values provide the following insights: as the value of Δδ increases, the chemical shift of LTTMs moves upfield, suggesting an enhancement in the electron density and a reduction in the hydrogen bond strength. Conversely, as the value of Δδ decreases, the chemical shift of LTTMs moves downfield, signifying a decrease in the electron density and reinforcement of the hydrogen bonding. The chemical shift of the –OH group of ChCl shifts upfield, which suggests a weakening of the hydrogen bonds that were initially present within the ChCl. The signals assigned to the γ-OH groups shift notably downfield compared to that of the 4-OH group, suggesting that the γ-OH groups preferentially created the hydrogen bonds during LTTM formation, leading to stronger interactions. Furthermore, the γ-OH group signal in E5 shifts significantly compared to that in E4, suggesting that E5 forms stronger hydrogen bonds. These results are consistent with the FT-IR results. The Δδ values of hydrogen atoms other than the –OH group at 25 °C are shown in Figure S11. Interestingly, the Δδ values of protons at OMe and 2-position (Figure S11a,b) do not change considerably before and after the LTTMs formation, suggesting that they do not contribute to its formation. In contrast, the chemical shift values at positions 5 and 6 of the aromatic ring (Figure S11c,d) exhibit a downfield shift and may contribute to the formation. The spectral variations at the positions agree with the C–H stretching shifts observed in the FT-IR spectra. Although the downfield shift was also observed for other protons (Figure S11e,f) in the ^1^H NMR spectra, a detailed study of these interactions is yet to be pursued. The Δδ values of the –OH group protons and the non–OH group protons of E5 and E4 at various temperatures were measured. The proton signals of the –OH groups show significant changes in Δδ with an increase in temperature in both samples (Figures 4a and 5a). On the other hand, the Δδ values for the non–OH group protons vary only slightly with temperature (Figures 4b–d and 5b–d). This finding supports the idea that the LTTMs form a hydrogen-bonding network,^39,40,45^ with varying hydrogen bond strengths.

**Figure 3:**
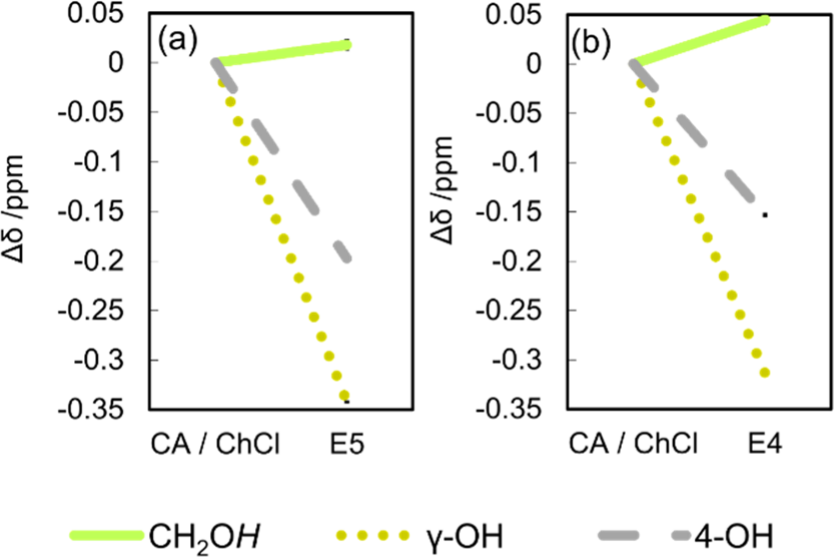
Δδ values of –OH groups of (a) E5 and (b) E4 at 25 °C (DMSO-*d*_6_ with 0.05% v/v TMS).

**Figure 4:**
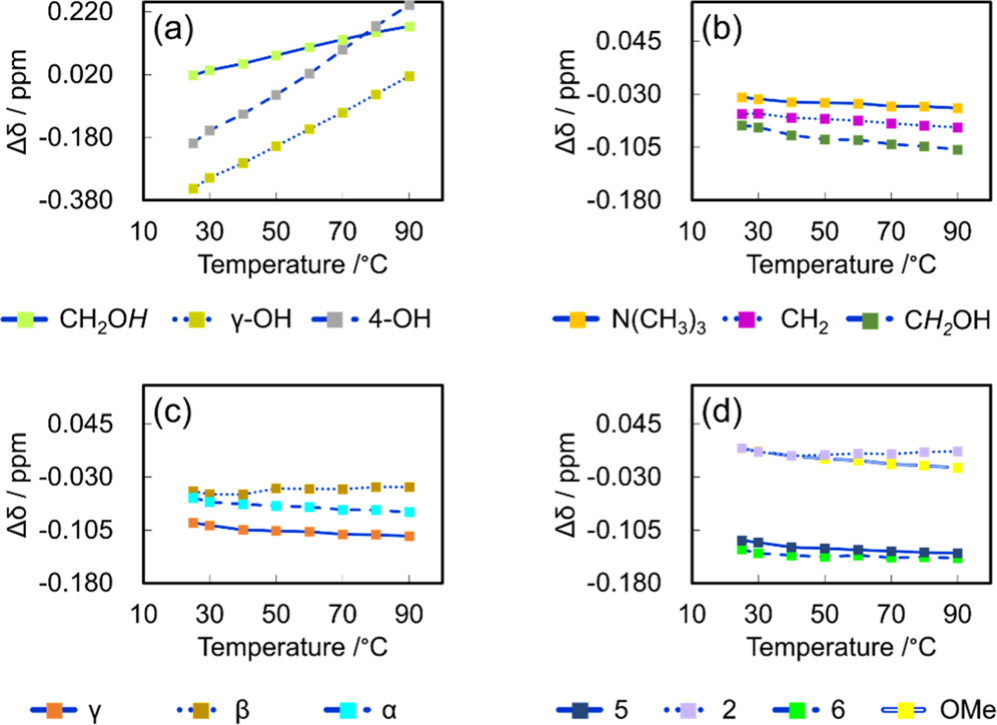
Δδ values of E5 for (a)–OH groups, (b) ChCl, (c) side chain of CA, and (d) aromatic ring of CA.

**Figure 5:**
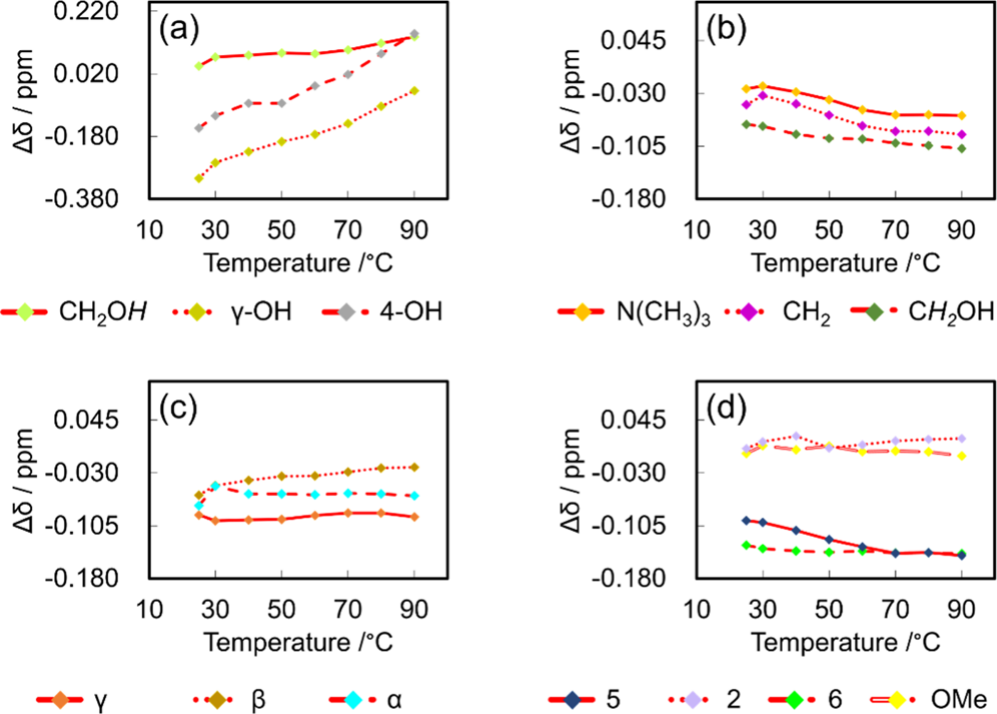
Δδ values of E4 for (a)–OH groups, (b) ChCl, (c) side chain of CA, and (d) aromatic ring of CA.

In summary, the temperature-dependent structural changes of the LTTMs prepared in this study are as follows: at lower temperatures, hydrogen bonds are formed among the –OH groups and, as previously reported, between the –OH groups and Cl ions,^46,47^ whereas as the temperature increases, weak hydrogen bonds such as those between CH–OH and those in the third and second solvation shells,^24,48,49^ gradually break down. Eventually, regions aggregated by hydrogen bonds and other regions coexist in approximately equal proportions.

In this study, the macrostructural and microstructural properties of CA-based LTTMs were investigated. E4 and E5 exhibited no discernible *T*_m_ values. The water content of E5 was higher than that of E4. The macrostructural analysis revealed that samples were composed of two distinct components at all temperatures. The FT-IR and ^1^H NMR results indicated that the γ-OH and 4-OH groups of CA interacted with each other or with the Cl ions to aggregate through hydrogen bonds, and other protons were not involved in such strong interactions. The temperature dependence of the structural changes was also explored in detail. As the temperature increases, strong hydrogen bonds between the –OH groups and between the –OH groups and Cl ions persist, whereas weaker hydrogen bonds, such as those between CH–OH groups and –OH groups located in the third and second solvation shells, gradually begin to break. For a more in-depth structural elucidation, advanced techniques such as neutron scattering, computer simulations, and ^35^Cl NMR are essential. These measurements may offer valuable insights into the interactions of non–OH protons, which were not elucidated in this study, and elucidate the rational relationship between the thermal transition temperature (*T*_m_ and *T*_g_) and the underlying hydrogen bonding network.

## Supplementary Material



## Notes

Additional GC–MS spectrum, NMR data, FT-IR spectra, and DSC curves data (PDF)

## Abbreviations

ATR: attenuated total reflection
Bet: betaine
CA: coniferyl alcohol
ChCl: choline chloride
COSY: correlation spectroscopy
CPMG: Carr–Purcell–Meiboom–Gill
DES: deep eutectic solvents
DMSO-*d*_6_: dimethyl sulfoxide-*d*_6_
DSC: differential scanning calorimetry
FT-IR: Fourier-transform infrared spectroscopy
HBAs: hydrogen bond acceptors
HBDs: hydrogen bond donors
HOESY: heteronuclear Overhauser effect spectroscopy
HSQC: heteronuclear single quantum coherence
LTTMs: low transition temperature mixtures
NMR: nuclear magnetic resonance
NOESY: nuclear Overhauser effect spectroscopy
TD-NMR: time–domain nuclear magnetic resonance
*T*_g_: glass transition temperature
*T*_m_: melting points
*T*_2_: transverse relaxation time
