# Supplementary material for: Structural Properties of Coniferyl Alcohol-Based Low Transition Temperature Mixtures
Source: ACS Omega. 2026 Jan 29;11(5):7501–7. doi: 10.1021/acsomega.5c08613 (PMC12902860; doi:10.1021/acsomega.5c08613)
Supplement: Supplementary file 1 [file ao5c08613_si_001.pdf]

Structural properties of coniferyl alcohol-based low transition temperature mixtures

Kosuke Ikeda [†], *Takumi Karasawa* [‡], *Takeki Miyazawa* [§], *Yoshiki Horikawa* [†], *Kento Kimura* [‡], *Yoichi Tominaga* [‡], *Toshiyo Kato* [‖], *and Yasuyuki Matsushita* [†,*]

[†] *Institute of Agriculture, Tokyo University of Agriculture and Technology, 3-5-8 Saiwai-cho, Fuchu, Tokyo 183-8509, Japan*

[‡] *Institute of Engineering, Tokyo University of Agriculture and Technology, 2-24-16 Naka-cho, Koganei, Tokyo 184-8588, Japan*

[§] *Faculty of Engineering, Tokyo University of Agriculture and Technology, 2-24-16 Naka-cho, Koganei, Tokyo 184-8588, Japan*

[‖] *Smart-Core-Facility Promotion Organization, Tokyo University of Agriculture and Technology, 2-24-16 Naka-cho, Koganei, Tokyo 184-8588, Japan*

* yasu@go.tuat.ac.jp

Figure S1 NMR spectra of CA; (a) ^{1}H NMR, and (b) ^{13}C NMR in DMSO-d_6 with 0.05 % v/v TMS

Figure S2 Mass spectrum of coniferyl alcohol measured by GCMS

Figure S3 NMR spectra of E5 at 25˚C; (a) ^{1}H NMR, and (b) ^{13}C NMR in DMSO-d_6 with 0.05 % v/v TMS

Figure S4 HSQC spectrum of E5 at 25˚C in DMSO-d_6 with 0.05 % v/v TMS

Figure S5 COSY spectrum of E5 at 25˚C in DMSO-d_6 with 0.05 % v/v TMS

Figure S6 NMR spectra of E4 at 25˚C; (a) ^{1}H NMR, and (b) ^{13}C NMR in DMSO-d_6 with 0.05 % v/v TMS

Figure S7 HSQC spectrum of E4 at 25˚C in DMSO-d_6 with 0.05 % v/v TMS

Figure S8 COSY spectrum of E4 at 25˚C in DMSO-d_6 with 0.05 % v/v TMS

Figure S9 The DSC carves of E1–5 at the second heating

Figure S10 IR spectra of E4 (red) and E5 (blue) in the 3800-2800 cm^{-1}

Figure S11 The Δδ values at non-OH groups; OMe and 2 of (a) E5, and (b) E4, and 5 and 6 of (c) E5, and (d) E4, and $N(CH_3)_3$, CH_2, $C\mathit{H_2}OH$, γ, β, α of (e) E5, and (f) E4 at 25 ˚C in DMSO-d_6 with 0.05 % v/v TMS

Table S1 Amount of H in CA–ChCl LTTMs per 1 mol of H in H_2O

Figure S1

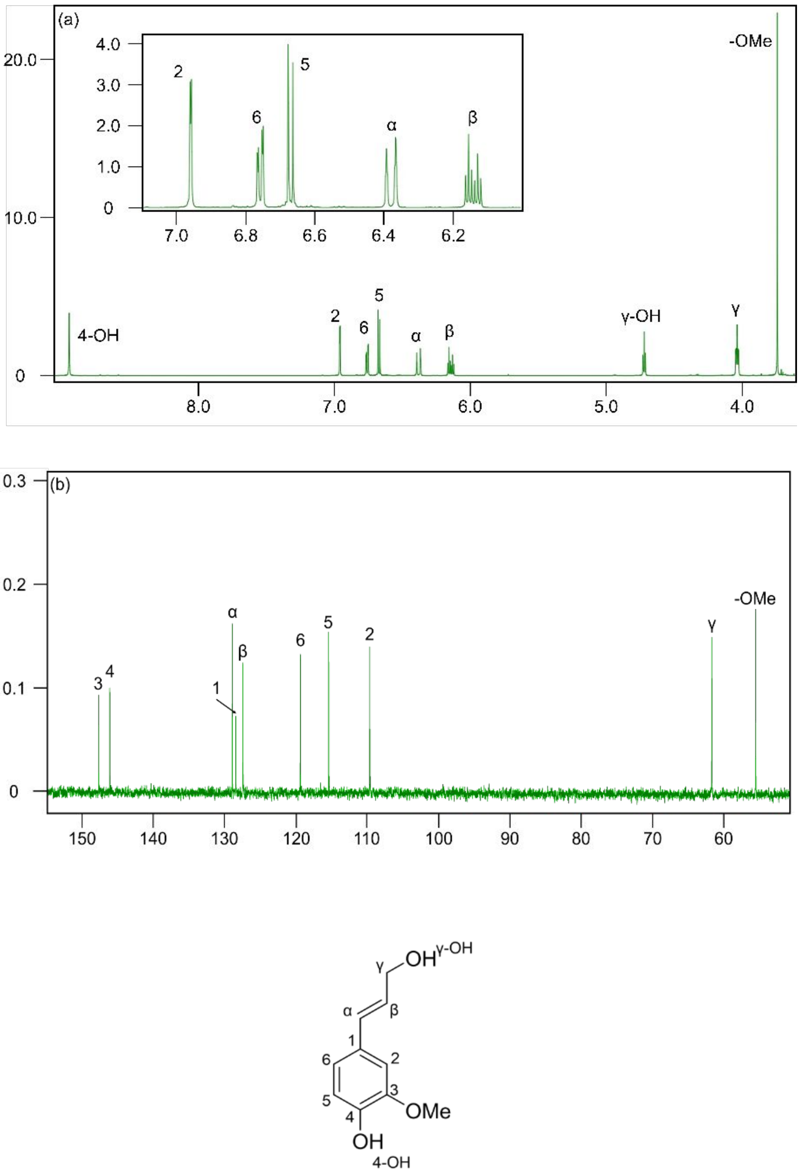

Figure S1 NMR spectra of CA; (a) ^{1}H NMR, and (b) ^{13}C NMR in DMSO-d_6 with 0.05 % v/v TMS

Chemical shifts (δ) are reported in ppm and referenced to the residual signal of DMSO-d_6 (δ = 2.49 ppm for ^{1}H, δ = 39.5 ppm for ^{13}C)

(a) ^{1}H NMR (DMSO-d_6 with 0.05 % v/v TMS, 600 MHz) δ: 3.77 (3H, s, OMe), 4.07 (2H, td, γ, J = 1.4, 5.5), 4.76 (1H, t, γ-OH, 5.5), 6.18 (1H, dt, β, J = 5.5, 15.8), 6.42 (1H, dt, α, J =1.4, 15.8), 6.71 (1H, d, 5, J = 8.3), 6.79 (1H, dd, 6, J = 2.1,

Figure S2

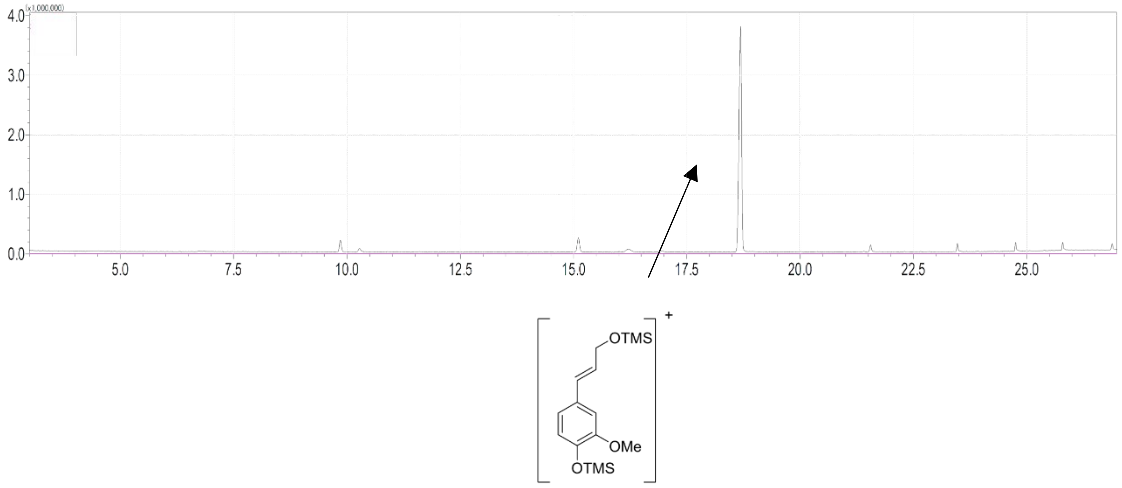

Figure S2 Mass spectrum of coniferyl alcohol measured by GCMS

Figure S3

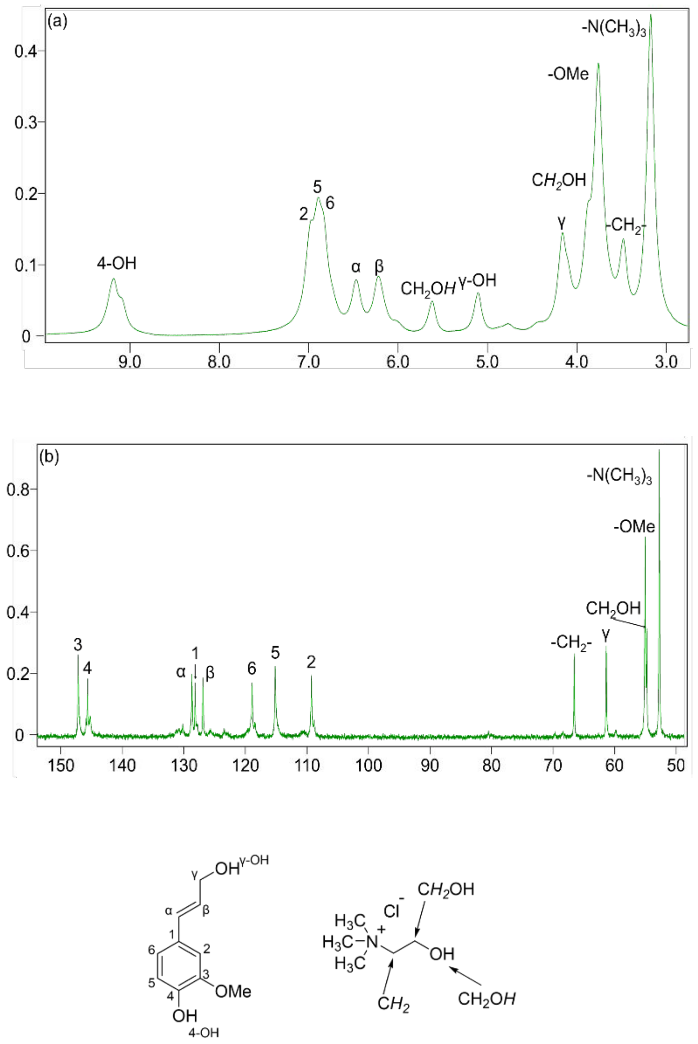

Figure S3 NMR spectra of E5 at 25˚C; (a) ^{1}H NMR, and (b) 13C NMR in DMSO-d_6 with 0.05 % v/v TMS

Chemical shifts (δ) are reported in ppm and referenced to the residual signal of DMSO-d_6

(δ = 2.49 ppm for ^{1}H, δ = 39.5 ppm for ^{13}C)

(a) ^{1}H NMR (DMSO-d_6 with 0.05 % v/v TMS, 600 MHz) δ: 3.17 (-$N(CH_3)_3$), 3.48 (-CH_2-), 3.76 (OMe), 3.86 (CH_2OH), 4.16 (γ), 5.11 (γ-OH), 5.62 (CH_2OH), 6.22 (β), 6.47 (α),

Figure S4

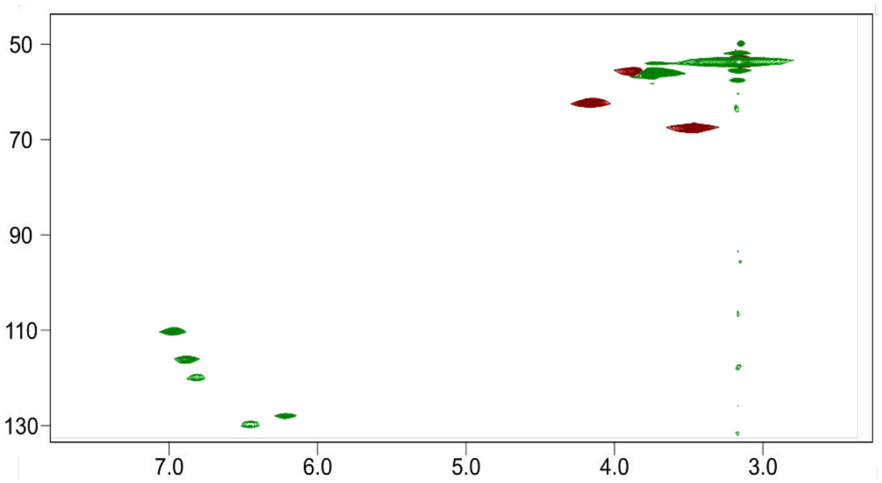

	δH	δC	HSQC
$-N(CH_3)_3$	3.17	52.7	$-N(CH_3)_3$
$-CH_2-$	3.48	66.5	$-CH_2-$
OMe	3.76	55.0	OMe
CH_2OH	3.86	54.7	CH_2OH
γ	4.16	61.3	γ
γ-OH	5.11	-	-
CH_2OH	5.62	-	-
β	6.22	126.9	β
1	-	128.1	-
α	6.47	128.7	α
4	-	145.6	-
3	-	147.2	-
6	6.83	118.9	6
5	6.89	115.1	5
2	6.97	109.3	2
4-OH	9.18	-	-

Figure S4 HSQC spectrum of E5 at 25˚C in DMSO-d_6 with 0.05 % v/v TMS

Figure S5

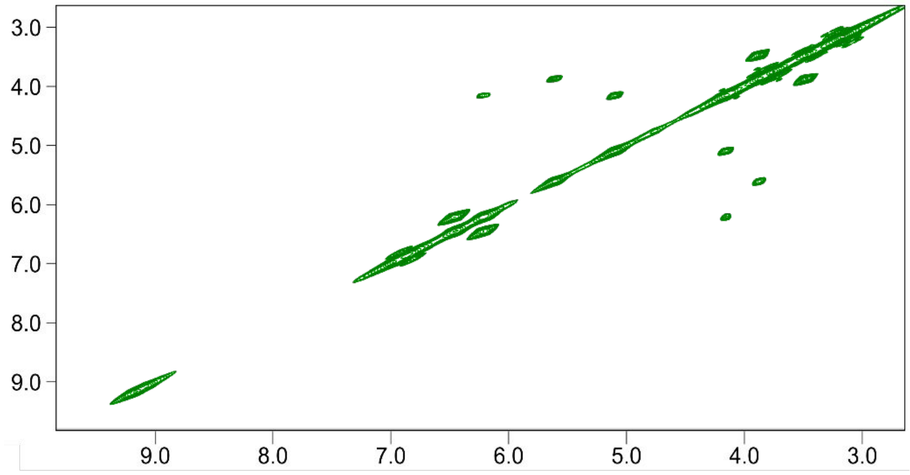

δHx	δHy	COSY
3.48	3.9	$-CH_2-/C\mathit{H}_2OH$
3.86	5.6	$C\mathit{H}_2OH/CH_2O\mathit{H}$
4.16	5.1	γ/γ-OH
4.16	6.2	γ/β
6.22	6.5	β/α
6.83	6.9	6/5
6.83	7.0	6/2

Figure S5 COSY spectrum of E5 at 25˚C in DMSO-d_6 with 0.05 % v/v TMS

Figure S6

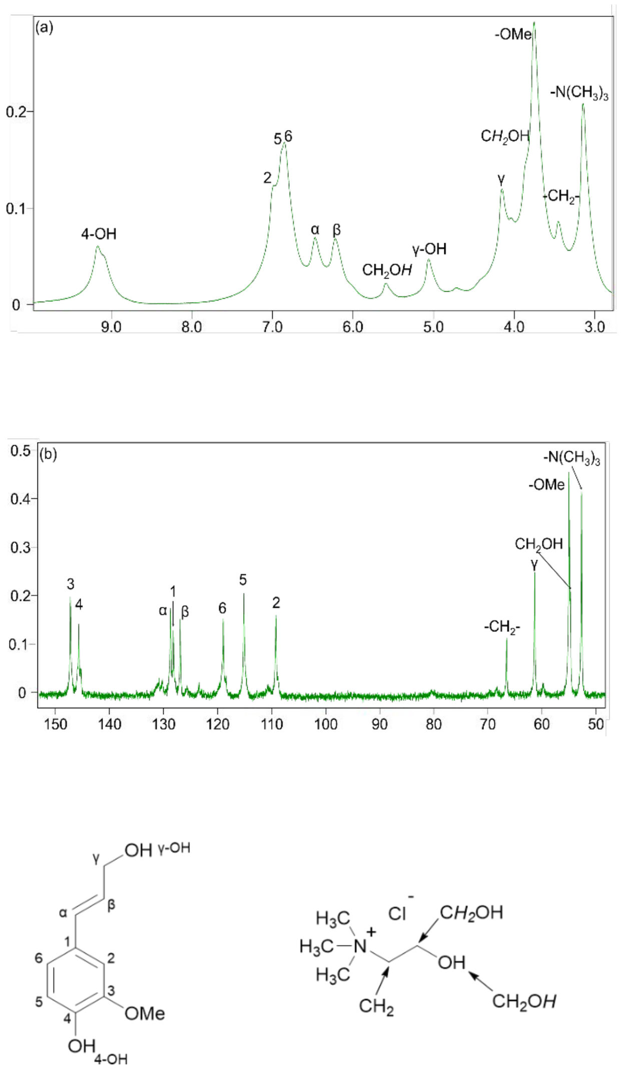

Figure S6 NMR spectra of E4 at 25°C; (a) ^{1}H NMR, and (b) ^{13}C NMR in DMSO-d_6 with 0.05 % v/v TMS

Chemical shifts (δ) are reported in ppm and referenced to the residual signal of DMSO-d_6

(δ = 2.49 ppm for ^{1}H, δ = 39.5 ppm for ^{13}C)

(a) ^{1}H NMR (DMSO-d_6 with 0.05 % v/v TMS, 600 MHz) δ: 3.16 (-N(CH_3)$_3$), 3.49 (-CH_2-),

3.77 (OMe), 3.86 (CH_2OH), 4.16 (γ), 5.07 (γ-OH), 5.60 (CH_2OH), 6.22 (β), 6.47 (α), 6.84 (6),

Figure S7

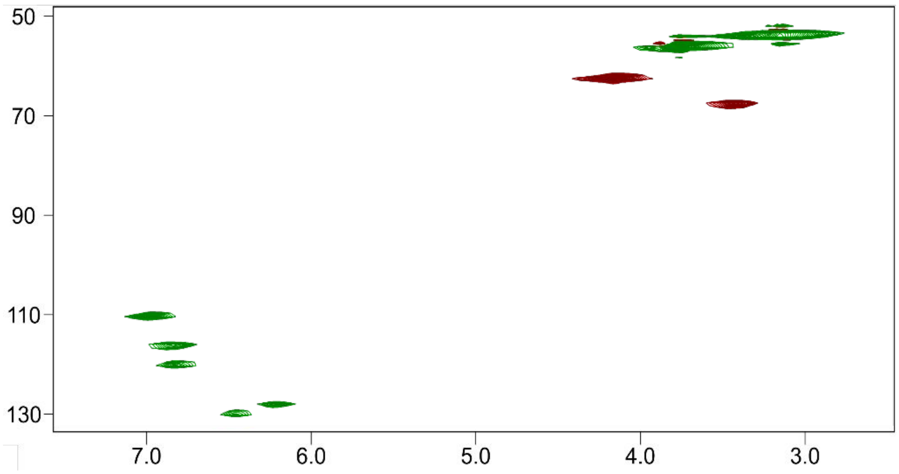

	δH	δC	HSQC
-N(CH_3)$_3$	3.16	52.7	-N(CH_3)$_3$
-CH_2-	3.49	66.6	-CH_2-
OMe	3.77	55.0	OMe
CH_2OH	3.86	54.8	CH_2OH
γ	4.16	61.4	γ
γ-OH	5.07	-	-
CH_2OH	5.60	-	-
β	6.22	126.9	β
1	-	128.2	-
α	6.47	128.7	α
4	-	145.7	-
3	-	147.2	-
6	6.84	118.9	6
5	6.88	115.1	5
2	6.98	109.2	2
4-OH	9.15	-	-

Figure S7 HSQC spectrum of E4 at 25˚C in DMSO-d_6 with 0.05 % v/v TMS

Figure S8

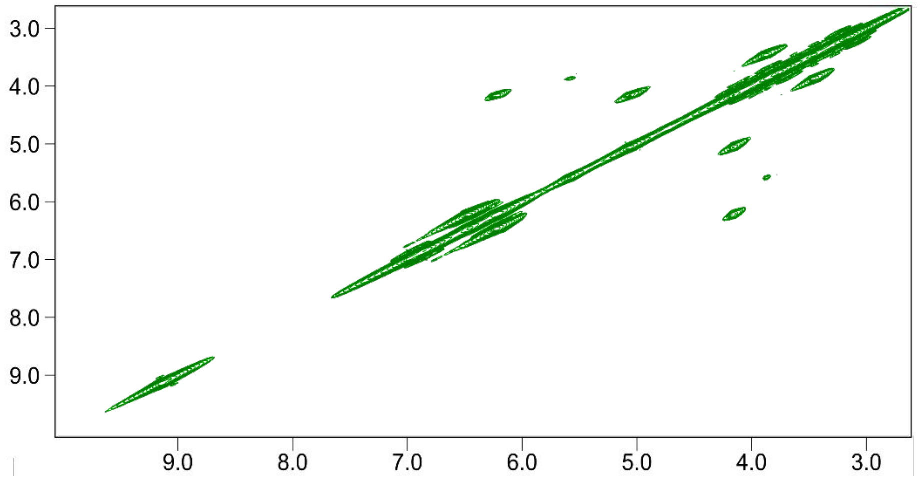

δHx	δHy	COSY
3.49	3.6	-CH_2-/C*H*$_2$OH
3.86	5.6	C*H*$_2$OH/CH_2O*H*
4.16	5.1	γ/γ-OH
4.16	6.2	γ/β
6.22	6.5	β/α
6.84	6.9	6/5
6.84	7.0	6/2

Figure S8 COSY spectrum of E4 at 25°C in DMSO-d_6 with 0.05 % v/v TMS

Figure S9

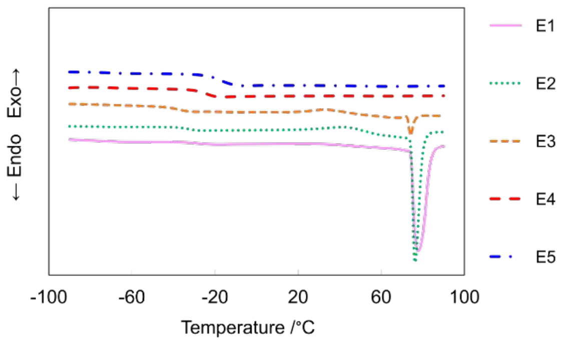

Figure S9 The DSC carves of E1–5 at the second heating

Figure 10

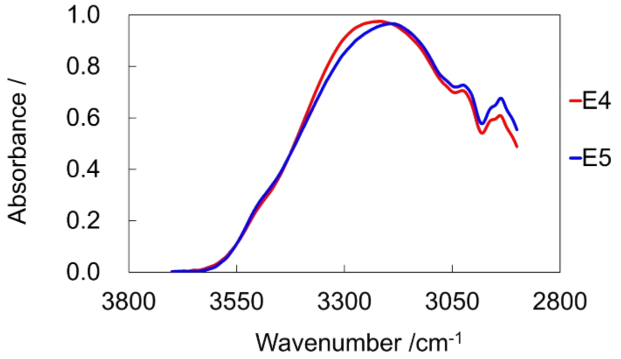

Figure S10 IR spectra of E4 (red) and E5 (blue) in the 3800-2800 cm^{-1}

Figure S11

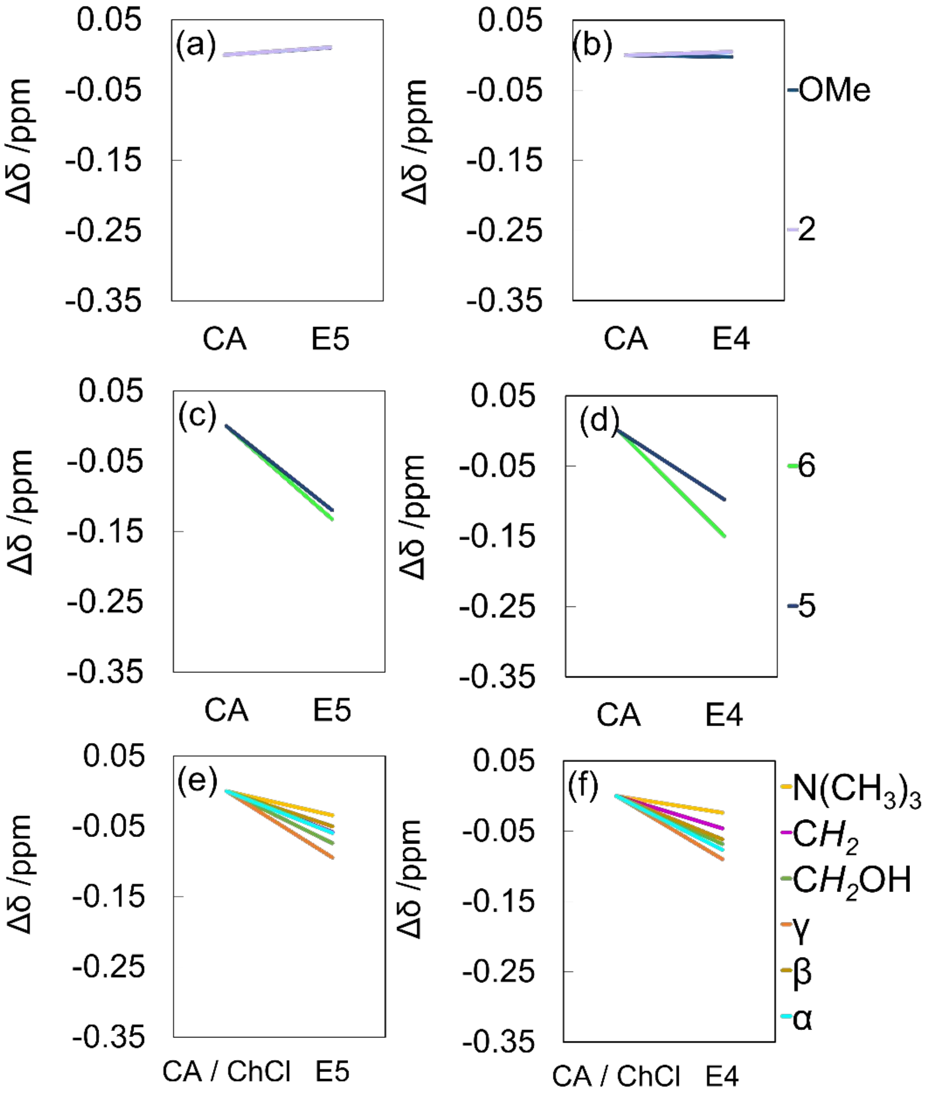

Figure S11 The Δδ values at non-OH groups; OMe and 2 of (a) E5, and (b) E4, and 5 and 6 of (c) E5, and (d) E4, and $N(CH_3)_3$, CH_2, CH_2OH, γ, β, α of (e) E5, and (f) E4 at 25 ˚C in DMSO-

Table S1

Table S1 Amount of H in CA–ChCl LTTMs per 1 mol of H in H_2O

CA:ChCl (molar ratio)	H_{CA} (mol)	H_{ChCl} (mol)	H_{CA} + H_{ChCl} (mol)	H_{H_2O} (mol)
2:1	90.188	52.610	142.797	1
3:1	61.940	24.088	86.027	1
